# Predicting protein ligand binding motions with the conformation explorer

**DOI:** 10.1186/1471-2105-12-417

**Published:** 2011-10-27

**Authors:** Samuel C Flores, Mark B Gerstein

**Affiliations:** 1 Department of Cell and Molecular Biology, Uppsala University, BMC Box 596, Uppsala, 75124, Sweden; 2 Department of Molecular Biophysics and Biochemistry, Yale University, PO Box 208114 MBB, New Haven, CT, 06520, USA; 3 Department of Computer Science, Yale University, PO Box 208114 MBB, New Haven, CT, 06520, USA

## Abstract

**Background:**

Knowledge of the structure of proteins bound to known or potential ligands is crucial for biological understanding and drug design. Often the 3D structure of the protein is available in some conformation, but binding the ligand of interest may involve a large scale conformational change which is difficult to predict with existing methods.

**Results:**

We describe how to generate ligand binding conformations of proteins that move by hinge bending, the largest class of motions. First, we predict the location of the hinge between domains. Second, we apply an Euler rotation to one of the domains about the hinge point. Third, we compute a short-time dynamical trajectory using Molecular Dynamics to equilibrate the protein and ligand and correct unnatural atomic positions. Fourth, we score the generated structures using a novel fitness function which favors closed or holo structures. By iterating the second through fourth steps we systematically minimize the fitness function, thus predicting the conformational change required for small ligand binding for five well studied proteins.

**Conclusions:**

We demonstrate that the method in most cases successfully predicts the holo conformation given only an apo structure.

## Background

Conformational changes in proteins can take place in a wide variety of ways, not all of which have been formally classified. One important class of motions is shear, in which stacked side chains of the protein can slide without losing contact. In this work we focus on the largest class, domain hinge bending, in which one structural domain of the protein moves relative to another domain about a hinge which connects the two [1,2]. Such motions typically involve the slowest degrees of freedom of that protein and so are difficult to predict by existing methods.

The prediction of ligand binding motions of the protein receptor has considerable potential applications in protein-protein and protein-ligand docking. Many methods can predict the side chain rearrangements required for docking [3,4] but these assume that the large scale conformation is already nearly correct. Thus there is a need for a method that will put the receptor in the correct large scale conformation which can be a productive starting point [5].

Much work has been done in this area. Molecular Dynamics (MD) [6-9] explicitly computes the dynamical trajectory of molecules modeled as point masses connected by linear and nonlinear springs and can be used to predict conformational change, but usually only small- or moderate-scale domain motions can be reproduced [10] with many biologically relevant motions remaining out of reach [11]. Accordingly several methods used MD to account for the fast fluctuations of proteins in drug docking by first computing the protein trajectory using MD [4,12,13]. One limitation of such techniques is that they may not escape the vicinity of an initial conformation, even in a time span experimentally known to be sufficient for conformational change [14]. Althaus et al created a combinatorial tree of side-chain rotamers which they explored using a branch-and-cut algorithm, [15] without varying the backbone conformation. Sandak et al. created a flexible-receptor docking code which articulates the protein at a hinge point, but leaves the two resulting domains rigid [16]. This method suffered from the opposite problem: it could generate large scale protein motions, but had no way of dealing with even small side chain rearrangements, a weakness leading to failure [15]. The described methods are good at either treating the side-chain flexibility, or the large scale conformational changes, but not both simultaneously. Conformation Explorer uses Sandak et al.'s idea of moving domains about a hinge point to generate large scale conformational change, but also includes equilibration steps which permit relaxation and adjustment of all atoms.

Normal modes have also been used by many authors to predict the conformational changes of proteins [17]. Comparison of the atomic coordinates of homologous pairs of proteins shows that the lowest order modes are most involved in conformational change, [18,19] but also that multiple modes are needed to accurately represent the motion [20]. It is possible to determine the correct combination of normal modes that will reproduce a desired motion, but this requires knowledge of at least a few interatomic distance constraints for the final structure [21].

In a different approach, a docked protein-ligand complex was displaced along the lowest-frequency normal mode directions to minimize non-bonded energy terms in an MD force field [22-24]. However a normal mode expansion assumes a quadratic potential and so is accurate only for small fluctuations about an equilibrium structure; therefore the method cannot be used to predict larger scale conformational changes such as we treat in this work. The method of Lindahl *et al*. gains improvements of 0.3 to 3.2Å for several proteins; [22] our method recapitulates much larger conformational changes as we will show.

Maiorov and Abagyan [25] rigidified all protein bonds except those in the interdomain linker and interface using Internal Coordinate Modeling, and then used the Biased Probability Monte Carlo protocol to generate potential alternate conformations of the protein. The method succeeded in generating a large number of alternate conformations, and some of these were somewhat similar to alternate conformations known crystallographically. However without referring to the known alternate conformations, it was impossible to determine which of the many predicted structures was thermodynamically plausible. Further, many energy evaluations and minimizations were expended in evaluating generated conformers which were later discarded. Lastly, it was not easy to know how long a thorough exploration of conformation space would take, and no clear way to restrict the search to a given region of interest. Our method is similar in several ways to Maiorov et al.'s, but also addresses these limitations.

In more recent work, de Groot *et al*. [26] showed they could find the holo conformations of several ligand-binding proteins. The method relies on tCONCOORD, [5] which determines flexible regions by analyzing hydrogen bonding networks. Once these are known, an ensemble of plausible structures is generated. An interative process involving docking, MD refinement, and filtering by radius of gyration then generates holo structures. However the radius of gyration must be provided by experiments, because their fitness function does not take into account the receptor conformation. Our work resembles de Groot's in the use of docking and MD refinement, but differs in that the hinge location is determined by the user (allowing the use of high-accuracy hinge detectors [27]), and in that it requires no experimental information about the holo complex. Our method is also computationally cheaper. Key to our method is the conformational sampling and the use of a fitness function *f *which includes terms to discriminate the holo conformation from the generated ensemble.

The mentioned *f *deserves motivation. There are numerous potentials which pick out the correct ligand-binding pose in a given receptor [28-30]. However these typically have no terms to discriminate favorable from unfavorable receptor conformations. Other force fields have been trained and tested against ensembles of crystallographically obtained protein structures, but it is not clear how they would perform when the *holo *structure is unavailable [31]. To overcome these limitations *f *includes terms (radius of gyration R_G_, stability E_F_, and docked energy G_D_) which help discriminate predicted *holo *complexes from a generated ensemble.

In this work we show that the Conformation Explorer can predictably, controllably, and economically generate multiple alternate conformations of proteins without experimental constraints [26] on the holo complex. The conformations can be equilibrated to ensure they are free of steric strain (close contact between atoms, or unnatural bond lengths and angles) [32].

We demonstrate that as an application of the method, we can predict the ligand bound, or holo, conformation of a protein given only the ligand and the coordinates of the protein in the apo state. The exploration of phase space is performed in such a way as to minimize *f*, which includes terms measuring the radius of gyration R_G_, stability E_F_, and docked energy G_D_. We will formally define all of these terms in the *Methods *section.

## Methods

As mentioned, the method involves the identification of the hinge and rotations about that hinge, followed by equilibrations and docking runs. The direction of the rotations and the choice of intermediate structures to which the rotations are applied, are subject to optimization. We describe how to perform these steps.

### Determining and recording the hinge location

The hinge can be detected in several ways:

#### Theoretically

Hinge predictors such as FlexOracle, [33] TLSMD, [34] HingeMaster, [27] and others [35-37] can be used to predict the hinge location if at least one structure is known. The HingeMaster web server makes the use of the first three easy [27] and was used in this work.

#### Crystallographically

If the protein has been crystallized in two different conformations, these can be inspected visually to determine the hinge location. This is usually the best and most accurate method [38].

#### By other experimental means

Proteolysis, [39] hydrogen/deuterium exchange, [40] optical trapping, [41] Fluorescent Resonance Energy Transfer (FRET), [42] Nuclear Magnetic Resonance, [43] and many other techniques can be used to determine the hinge location.

### Assignment of domains and calculation of centers of mass

**Figure 1:**
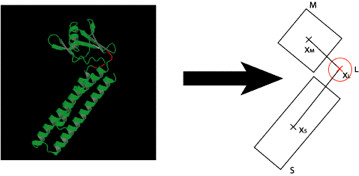
**Assignment of residues to S, L, and M**. The hinge residues are highlighted in red on the left. Residues from the first (N-terminal) residue to the lowest-numbered hinge residue belong to domain S. Residues following the first hinge region, but before any second hinge region, belong to domain M. Residues following the second hinge region (but before a third hinge region, if there is one) belong to S, and so on.

Our method relies on the identification of a "stationary" domain S (equivalent to Maiorov et al.'s domain A [25]), a mobile domain M (equivalent to Maiorov et al.'s domain B), and a linker or hinge region L (Figure 1). The linker can be single, double, or triple stranded, and the two domains can be continuous or discontinuous [44]. Each residue is assigned to S, M, or L. The centers of mass (COM's) [45] of S, M, and L are labelled X_S_, X_M_, and X_L_, respectively. These domain and COM definitions are used in the subsequent preparation and manipulation of the structure.

### Preparation of protein and ligand

**Figure 2:**
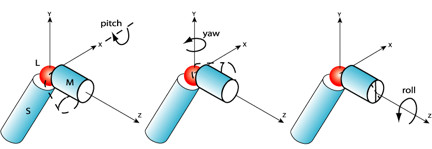
**Applying Euler rotations to M domain**. Rotations are applied about the z-axis, R_z_(θ_z_)_, _about the x-axis, R_x_(θ_x_), and about the y-axis, R_y_(θ_y_), in that order. The explored space of rotational orientations of the M domain spans ± 80° in the y and z directions and -20° to 80° in the x-direction. Rotations about x, y, and z axes (R_y_(θ_y_), R_x_(θ_x_), and R_z_(θ_z_)) result in putative alternate conformations. The standard orientation described in the text is such that the center of mass (COM) of L is at the origin, COM of M domain is along the z-axis, and COM of S is in the negative-y part of yz plane.

The Conformation Explorer at this time can handle only single protein chains. Therefore all additional peptides, ligands, metals, water, and dissolved ions are removed. The ligand of interest is docked to the structure using AutoDock. The entire receptor is in the grid map; we do not prejudice the docking with binding cleft information. The simulation is not sensitive to this initial docking, since there are subsequent *re-docking *steps as we will explain. It is important for the ligand to be docked before the equilibration (explained shortly), so that the latter does not lead to side chains filling in the binding cleft and and otherwise blocking the ligand from re-docking [17,46,47]. The protein is then put into *standard orientation*. This is the convention that X_L _coincide with the origin, X_M _lie along the z-axis, and X_S _lie in the -y part of the yz plane (Figure 2).

The coordinates of the ligand are required in PDB format, as is the GROMACS .itp (include topology) and the AutoDock .pdbq (structure + charges + bond mobility) files. The latter two can be generated from the first using the PRODRG program [48].

### Definition of Rotations

The three Euler rotations mentioned earlier are the space-centered rotation of the M domain about the z-axis, R_z_(θ_z_)_, _about the x-axis, R_x_(θ_x_), and about the y-axis, R_y_(θ_y_), applied in that order. (Figure 2). The θ_z_, θ_x_, and θ_y _are the angles of the corresponding rotations. The ligand is not rotated, i.e. remains stationary with respect to S.

### Equilibration

The preceding rotation step invariably results in unphysical bond lengths and angles in the boundary between L and M, and often in steric clashes between M and the rest of the protein and/or ligand. A Molecular Dynamics equilibration is performed using the GROMACS mdrun program for 3000 time steps (6 ps of simulation time). We solvated using a water box with a neutralizing atmosphere of Cl^- ^and Na^+ ^ions. We found that 1000 time steps (2ps) were sufficient to relieve the most significant steric strains, and 10000 time steps were sufficient to allow an exponential decrease in enthalpy to level off (data not shown), indicating a better equilibrated structure. It was not sufficient time to allow substantial domain motions. Equilibration significantly increased the accuracy of ligand-binding prediction, compared to what we got with no equilibration.

### Angle calculation

We calculate the angular position of M of a generated structure in θ_y_θ_x_θ_z _space by determining the R_y_(θ_y_)R_x_(θ_x_)R_z_(θ_z_)_, _rotation that would have to be applied to M of the starting structure in standard orientation, to obtain a structure similar to the generated one. This is done by first structurally aligning the generated structure with the *starting structure *by minimizing the root-mean-square deviation (RMSD) between the S domains of the two structures. The θ_y_, θ_x_, and θ_z _angles are then computed. This calculation is performed immediately following each equilibration step above. Note that in the course of the equilibration the angular position of M will change. We refer to this as *drift*.

### Re-docking

At the end of each equilibration the ligand is removed from the structure file and then docked again to the protein using AutoDock 3 [49]. The docking code reports multiple poses (docked ligand atomic positions) with corresponding free energies of binding; we record the lowest of the latter as the G_D_, as we discuss below. The corresponding ligand coordinates are then appended to the protein structure file.

Terms of *f*: G_D_, R_G_, E_F_

The fitness function *f *we devised to discriminate the holo structure from the many conformations generated includes four quantities which separately have strengths and weaknesses, but which together form a predictor. In this section we describe the terms, which are combined in a weighted sum to create *f*.

G_D_, as mentioned, was computed using AutoDock at the end of each equilibration. This quantity was found early on to have significant ability to discriminate between near-holo structures and decoys. However in some cases an unnatural cleft was generated by the mentioned rotations, to which the ligand docked with high affinity. Such unnatural conformations often have large radii of gyration and can be filtered out on that basis. We also found that this measure did not vary smoothly with sRMSD (the RMSD between M domains of two proteins, after they have been optimally superimposed on their S domains) [25], thus no gradient was available along which to minimize. For these reasons we added terms to *f *as follows.

R_G _typically decreases during the cleft closure often associated with ligand binding, as has been observed by Small Angle X-ray Scattering (SAXS), [50-52] and as we will further demonstrate here. This quantity is the distance *Rg *from the center of mass at which all the mass of the protein's C_α _atoms can be concentrated to result in the same moment of inertia: [53]

(1) $$Rg=\sqrt{\frac{\underset{i}{\sum }mr_{i}^{2}}{M}}$$

Where *i *counts over all atoms, *r_i _*is the distance (in Angstroms) from the atom to the center of mass, and M is the total mass. It also varies smoothly with sRMSD, and so helps deal with the noise issues mentioned above and leads to improved convergence. However R_G _alone is not a good predictive measure because it is trivially possible to minimize it with an unstable protein structure consisting of distorted interpenetrating domains. This problem led to the introduction of the next quantity into *f*.

*Energy of folding*, or E_F_, can be used to discriminate energetically feasible conformers from those with excessive interpenetration or unnatural orientation of domains. This quantity is estimated using FoldX. The latter is a force field empirically fitted by Guerois et al. using a database of mutationally induced changes in protein stability [54]. However since a wide variety of conformers, including both the holo and the apo, are stable, this measure alone cannot find the holo structure in an ensemble. The point of computing this quantity is again to exclude unphysical structures.

### Figure of merit: sRMSD

Once the alternate conformers have been generated by rotation, equilibration, and re-docking, we are faced with the problem of determining how distant these are from the known target conformer, in our case the protein from the co-crystallized complex. For this purpose Maiorov et al. used a "static" root-mean-square deviation (sRMSD), defined as the RMSD of the Cα atoms in M, given that the Cα atoms of S are optimally superimposed. We use the same measure in the current work.

### Five-fold leave-one-out cross-validation of *f*

We computed G_D_**, **E_F_, and R_G_^2 ^for every structure in each of the five generated ensembles (corresponding to the five proteins). We then divided the proteins into a single *test *protein and a *training set *consisting of the remaining four proteins. We did this five times, choosing a different *test *protein each time. Each time, *f *was fitted to the *training set *as follows.

We first define *f *as:

(2) f=xλ

Where *x *is a matrix with one row for each structure in the *training set *and one column for each of the quantities G_D_**, **E_F_, and R_G_^2^. The element in a given row and column is the physical quantity corresponding to that column, computed on the structure corresponding to that row. *λ *is a three-element column vector of weights to be fitted as follows.

We assert that:

(3) xλ≈y

Where *y *is a column vector with each row containing the quantity sRMSD, computed for the structure corresponding to that row. Rows in *y *correspond to rows in *x*.

*λ *is then given by: [27]

(4) $$\lambda ={\left(x^{T}x\right)}^{-1}x^{T}y$$

Five different *λ*'s were computed in five-fold leave-one-out bootstrapping, [27] with one *λ *used for each protein, except where noted below.

### Holo, predicted, apo, and infeasible structures

**Figure 3:**
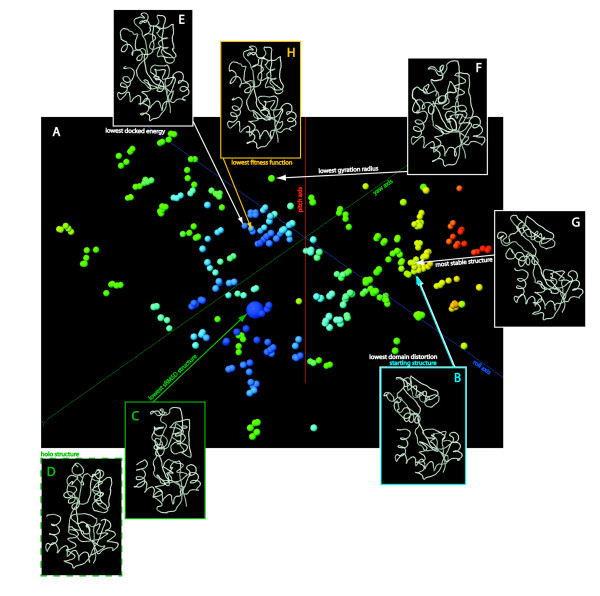
**Glutamine binding protein**. Conformation Explorer provides a jmol viewer (A) which represents each rotated, equilibrated, and redocked structure as a single sphere, with the position of each sphere representing the θ_y_, θ_x_, θ_z _rotation angle of domain S with respect to that of the *starting *structure (B), as explained in the text. We have colored the spheres by the *sRMSD*. The structure that minimizes *sRMSD *is in inset (C) and the *holo *structure is in (D). The *fitness function *includes *docked energy*, *gyration radius*, and *foldx energy *(G_D_**, **R_G_**, **E_F_) terms, and the structures that minimize these are show in E-G.

For each protein studied, we discuss three protein-ligand configurations (Figure 3). The holo structure is the crystallographically determined structure of the enzyme bound with ligand, which we use as a gold standard, and which would presumably be unknown in a practical situation. The apo structure is the protein structure which would be known in practice and which is used as a starting point in our analysis. The generated ensemble is the set of all structures generated by rotating M, equilibrating and docking. The *predicted *structure is the conformer chosen from the generated ensemble as the best approximation of the holo structure. In the ligand docking example explained here, it is selected based on the described *f*. An *infeasible *structure is one that could not be generated for one of two reasons: (1) the rotation was effected but the steric strains generated were too large and so the equilibration step failed to converge, or (2) the equilibration converged but the drift left the structure not in the targeted *cell *(we will define this term below) but in a neighboring one, even after two successive attempts at rotation and equilibration. In both cases the rotated but unequilibrated structure in the target cell was marked as *infeasible*. This marked the corresponding cell as being on the boundary of the accessible conformation space and prevented further attempts to generate conformers in it.

### Minimization of *f *by line search algorithm

We minimized *f *using the line search algorithm, which successively varies each of the angles (θ_y_, θ_x_, θ_z_) over its full range. After each angle is varied, the conformer that minimized *f *over the full range of that angle is used as a starting point for the next minimization. The sampling of θ_y_, θ_x_, θ_z _characteristic of this method can be appreciated graphically in Figure 3 (lines are not straight due to *drift *as discussed). The line search minimization can be considered converged when no R_y_(θ_y_), R_x_(θ_x_), or R_z_(θ_z_) rotation from the lowest-scoring conformer can further reduce the value of *f*. The algorithm is summarized in Additional File 1. In the following sections we describe the proteins we used for training, testing, and applying *f*.

### Glutamine binding protein (GlnBP)

The motion of GlnBP [27] as it binds glutamine involves large-displacement domain hinge bending, experimentally determined to take ~5ns [14] 6 ns Molecular Dynamics simulations (requiring 42 days on a dual-processor machine) of the apo structure failed to result in domain closure, possibly in part because the dynamics of the apo structure were computed with no ligand present [14]. The apo or starting structure used, [55] and the holo structure [56] both originate in *E. coli*.

For GlnBP FlexOracle [33] found a hinge at residues 86-89 and 182-185. We selected residues 88,89,181, and 182 as the hinge location for the purpose of generating the rotations, but since the flexure occurs at the boundary between L and M, this adjustment makes little difference.

### Biotin Carboxylase

Acetyl-CoA carboxylase (ACC), found in all animals, plants, and bacteria, catalyzes the carboxylation of acetyl-CoA to malonyl-CoA, the first committed step of fatty acid synthesis. The first half-reaction is the formation of carboxybiotin which is catalyzed by the Biotin carboxylase (BC) subunit [57].

Pyruvate carboxylase (PC) is found in many eukaryotes and some prokaryotes. It plays a role in gluconeogenesis, mediating the carboxylation of pyruvate to oxaloacetate. It has three functional domains, of which biotin carboxylase (BC) is one. The half-reaction catalyzed by BC is common to ACC and PC, although the second half-reaction catalyzed by a different subunit differs from enzyme to enzyme depending on the substrate [58]. Upon binding ATP, M rotates approximately 45° with respect to S. The large scale of this change and the existence of structural domains makes BC a good test case for our method.

We used the BC subunit of *Aquifex aeolicus *PC [58] as the starting or apo structure. The ligand-free BC subunit of *E. coli *ACC has been crystallized (PDB ID: 1DV1), but many residues are disordered and so the structure of the M domain is not entirely clear [59]. No ATP-bound structure of *A. aeolicus *BC is available, and so we used ATP-bound BC from *E. coli *ACC [59] as our holo structure. The sequence differences between the starting and holo structures had an impact on the results as we will discuss. FlexOracle [33] predicts a hinge at residues 86-89 and 182-185; this can be used to choose the location of L.

### Ribose Binding Protein (RBP)

RBP is a member of a large family of bacterial periplasmic binding proteins, [60] and displays clear domain hinge bending motion [27]. It has a triple stranded hinge, with a small fragment of the N-terminus crossing over onto the M-domain. It binds ribose, a small, oxygen-rich ligand which we will discuss in more depth later.

### Adenylate Kinase (ADK)

**Figure 4:**
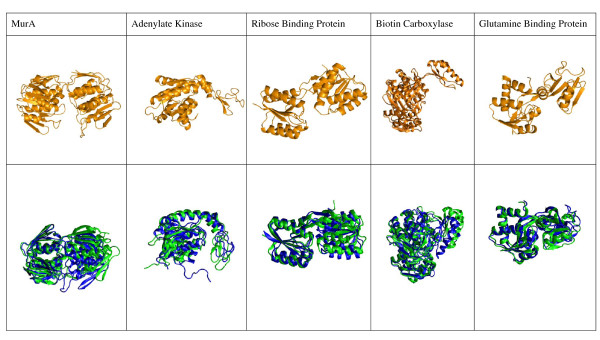
**Predicted conformational change**. The *apo*, or *starting *structure used as a starting point for the calculation is shown in orange on the top row. The M domain of this molecule is rotated with respect to the S domain until converging to the predicted structure (blue trace, bottom row). These generally align well with the known *holo *structures (green cartoons, superimposed on the blue, bottom row). Molecules are displayed such that the S domain is in the right-hand portion of each molecule, the M domain is in the left-hand portion, and the hinge is between them, with the binding cleft opening downwards. The predicted and *holo *structures are aligned based on the S domain (as for the sRMSD calculation) to highlight the degree of alignment of the M domains.

Adenylate Kinase is a popular example of a domain hinge bending protein (Figure 4). Even though the *holo *and *starting *structures come from the same organism, they are from different compartments and have significant structural differences. The *holo *structure was extracted from the mitochondrial intermembrane space, while the *starting *was taken from the mitochondrial matrix (Table 1). It naturally binds Mg-ATP and AMP [61], but it has been crys-tallized with SO_4 _(PDB ID: 1AK2).

**Table 1:** Results of five-fold leave-one-out cross-validation.

				sRMSD (Å)			
		
Protein	*apo* PDB	*holo* PDB	ligand	starting	lowest in ensemble	predicted holo	server ID
							
*RBP*	*1URP*	*1DRI*	*ribose*	*12.1*	*7.0*	*14.8*	*ce828024-27360*
*ADK*	*2AK3*	*1AK2*	*SO4*	*17.0*	*3.8*	*11.2*	*ce829841-12334*
RBP	1URP	1DRI	ATP	12.1	3.5	5.8	ce105518-17825
ADK	2AK3	1AK2	ATP	17.0	4.6	5.3	ce131413-8235
GlnBP	1GGG	1WDN	glutamine	15.7	4.2	5.3	ce963091-2997
BC	1ULZ	1DV2	ATP	14.8	6.7	10.9	ce889203-5498
MurA	1EJD	1A2N	T6361	4.9	4.8	9.0	ce336773-19756

The five proteins (RBP, ADK, GlnBP, BC, and MurA) were each submitted to CE. For each protein, the parameters of *f *were fitted using the results of the other four proteins. In a preliminary round we computed closure of RBP binding its natural ligand, ribose, and of ADK binding SO_4 _(with which it has been cocrystallized in *holo *form) but sRMSD of the predicted structures was high (14.8 and 11.2Å, italic type). In the final round of calculations these two proteins were submitted with ATP as a ligand (next two rows, regular type), resulting in much lower sRMSD. GlnBP and BC were submitted with their natural ligands, and MurA with an antibiotic.

The motion of ADK involves not two domains but three. Here we predicted the motion of the LID domain (residues 121-158) with respect to the CORE domain (residues 1-24, 81-118, 161-214). Since the AMPbd domain (residues 27-78) moves separately, for computing sRMSD we aligned based only on the CORE domain.

### MurA

MurA, which has been cyrstallized bound to the antibiotic T6361 [62]. The peculiarity of this ligand is that it binds to the open conformation of MurA, rather than the closed, and thus interferes with the mechanism of motion [62]. In this case if the predicted bound conformation is correct, it should not differ significantly from the open structure by the measure of sRMSD. The starting structure used was taken from the complex of MurA with an analogue of its natural ligand [63].

### ABC transporter periplasmic ligand-binding protein (ABC-PBP)

ABC-PBP is a very sparsely characterized protein. Its natural ligand is unknown, and few other details are available about its function [64]. It has been crystallized in the *apo *form (PDB ID: 3n0w). These characteristics make the protein a typical case in which a motion predictor like CE would be useful. In this work we make a blind prediction of its *holo *conformation.

## Results

### Natural vs. non-natural ligands

In a first fitting, we docked each receptor with the ligand it was crystallized with in *holo *(table 1). We observed that for two of the proteins (RBP and ADK), the ligand penetrated deeply into one of the two domains rather than binding at the cleft between the two. Observing that in both cases the ligand was small and oxygen-rich (ribose and SO4, respectively), we hypothesized:

1. That the docking is not effective for any small and oxygen-rich ligand.

2. That the precise choice of ligand is unimportant for the objective of predicting closed structures.

We ran CE on GlnBP with *non-natural *(i.e. other than glutamine) ligands (Table 2). We found that the three highest sRMSD's were obtained for SO4, ribose, and oxalic acid, the three small oxygen-rich ligands. In the three cases the ligand penetrated one of the two domains rather than binding in the cleft. (possibly because the docking force field insufficiently penalizes desolvation). Finding that ATP yielded the lowest sRMSD's, we ran CE on all five proteins using ATP. We found that in the cases of RBP and ADK, the sRMSD was lower than with their natural (small, oxygen-rich) ligands. These two results support hypothesis (1). We also found that for GlnBP and MurA, the results were slightly worse using ATP than the natural ligand, though only by an average of 1.5Å. Thus the specific ligand matters, but only slightly. In our final result we use ATP in place of SO4 and ribose, but make no such substitution when the natural ligand is not small and oxygen-rich (table 1).

**Table 2:** Results of running CE on GlnBP with various ligands.

legend	molecular weight	sRMSD (Å)
glutamine	146	4.56
leucine	146	6.07
cAMP	328	4.98
ATP	503	4.39
T6361	661	5.11
ribose	150	8.80
oxalic acid	88	7.31
SO_4_	96	12.37

The three small, oxygen-rich ligands (ribose, oxalic acid, and SO_4_) led to the worst results, because the ligands bound deep inside one of the two domains rather than at the cleft. Other ligands led to results comparable to those of glutamine.

### Glutamine binding protein (GlnBP)

We ran CE on this protein to demonstrate the online results browser, and to rationalize the three terms of the fitness function.

Figure 3.a shows each of the conformers (represented as spheres), positioned according to the angular orientation of Domain M, and colored by sRMSD. The conformer with lowest sRMSD is shown with a larger sphere than the others. The *starting structure *is indicated with the blue arrow, and displayed in inset 3.b. As mentioned *sRMSD *is our figure of merit for scoring our predictions. The generated structure with lowest sRMSD is shown in inset 3.c. The reader can verify that this structure is qualitatively similar to the experimentally known *holo structure*, shown in inset 3.d.

The structure with lowest G_D _is shown in inset 3.e, and is also qualitatively similar to the *holo*.

The structure with lowest *gyration radius *is shown in inset 3.f. The *holo *tends to have a smaller R_G _than the *apo *structure. R_G _cannot be used alone in *f *since it is trivially possible to minimize this quantity with a compact structure which is unstable, has significantly distorted domains, and does not resemble the *holo*. The *gyration radius *appears squared for the physical reasons mentioned earlier.

The structure with most favorable E_F_, inset 3.g, is quite close to the *starting structure*. This should not be surprising, since such structures have gone through a minimum of structural manipulation and so have low steric strain.

### Biotin Carboxylase

The significant sequence and structural differences between the *starting *and *holo *structures, which come from different organisms, made comparison on the basis of sRMSD somewhat difficult. The structure that minimizes *f *(Figure 4) bears clear similarities to the *holo *in relative domain orientation.

### Ribose Binding Protein (RBP)

In Figure 4, the structure that minimizes *f *with ATP as a ligand shows good agreement with the *holo*. The natural ligand led to poorer performance as mentioned.

### Adenylate Kinase (ADK)

The *starting *structure had 17Å, while the predicted structure (with ATP docked) had 5.3Å sRMSD (Table 1) - almost 10 Å lower. The qualitative agreement was excellent, as can be appreciated in Figure 4. On the other hand docking SO_4, _a ligand it has been crystallized with, led to poor results.

### MurA

We have now shown that the Conformation Explorer can predict the bound conformation for four cases in which a large scale closing motion is required. But what if the ligand binds to the open conformation? One such case is that of MurA. The predicted structure is more closed than the *holo*, indicating that *f *is biased towards closed structures (Figure 4).

### ABC-PBP

**Figure 5:**
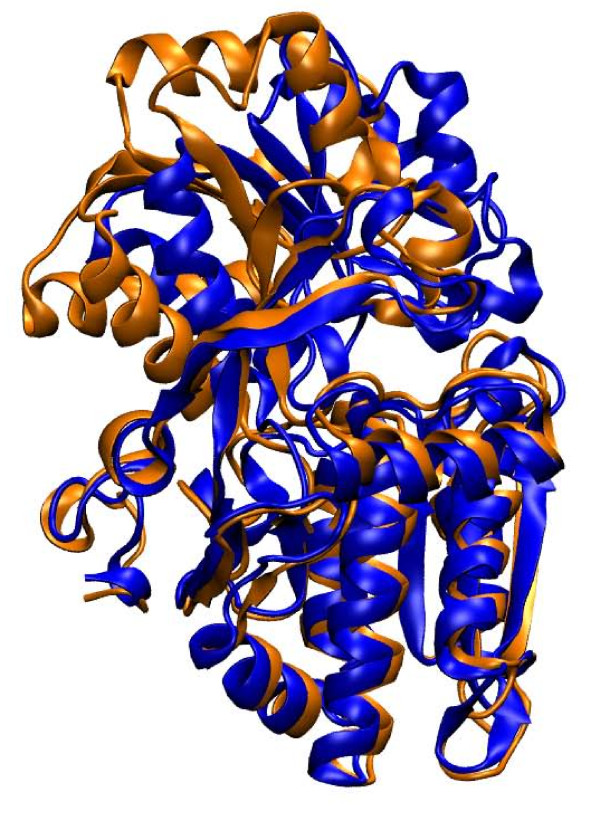
**Predicted ligand-binding motion of ABC-PBP**. ABC-PBP is not well characterized. We started from its *apo *structure (PDB ID: 3n0w, in gold) and generated a predicted structure (ce703645-18211, in blue). Validation is left to experimentalists.

The above five proteins are well characterized, with a known *holo *complex, unlike cases of scientific interest for which CE is useful. In order to demonstrate a practical application, we submitted ABC transporter perplasmic ligand binding protein (PDB ID: 3N0W) to CE. Since the natural ligand is unknown, we used ATP. The predicted structure is shown in Figure 5. We await future experimental validation of the predicted coordinates, which can be downloaded from MolMovDB.

### Fitness function

As mentioned in generating the predicted structure for each of GlnBP, BC, ADK, RBP, and MurA, we trained on the remaining four proteins. The final fitness function, trained on all five proteins, is:

(5) $$f=0.35\cdot G_{D}+0.066\cdot E_{F}+0.030\cdot R_{G}^{2}$$

### Computer time

Computational cost ranged from about 6 hours (for GlnBP) to 12 hours (for RBP) on a single processor. For comparison, the method of Seeliger *et al*. [26] requires about four days on a 50-node cluster.

### Web server

**Figure 6:**
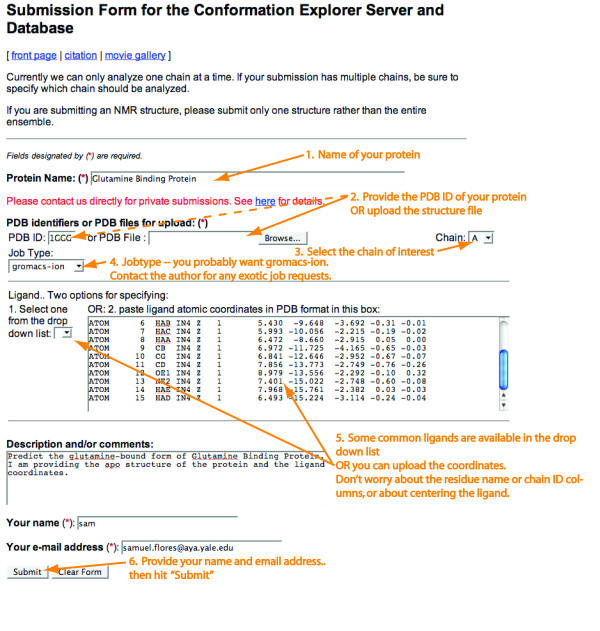
**Submission form for the Conformation Explorer server**. Note that for the receptor file, the user may provide a PDB ID or upload a file. For the ligand, there is a drop down list of several frequently used ligands, or the user may paste the ligand atomic coordinates in PDB format, in the text box. After all sections are filled in, click Submit. You are not done yet, however - the hinges must still be selected in a subsequent step.

**Figure 7:**
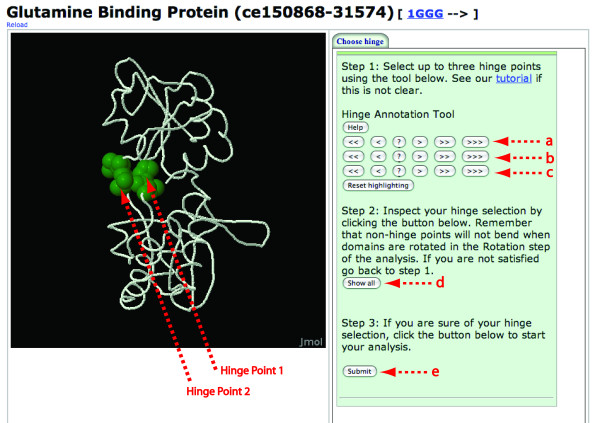
**Selecting the hinge points**. The Hinge Annotation Tool is used to select the hinge points. a. Use the first row of arrow buttons to select the first hinge point. Single arrow buttons (>/<) increase/decrease the hinge selection in single-residue increments. Double arrow buttons (>>/<<) increase/decrease the selection in 10 residue increments. The triple arrow button (>>>) increases in 100 residue increments. Use the "?" button if you are not sure what residue number is currently selected. Use the second row of arrow buttons, select hinge point 2, if there is one. If the hinge is triple stranded, use the third row for the third hinge point. Show all hinges to verify your choices. Click "Submit" when done. You will be asked to confirm your submission, then your job will be queued.

The described tool can be used through our online server at http://MolMovDB.org. The user must first provide the structural coordinates of the receptor and ligand, as described in Figure 6. He/she must then select the hinge points. For this we make use of the Hinge Annotation Tool on the Database of Macromolecular Motions http://MolMovDB.org morph page, as described in separate work [33,65]. Briefly, it consists of arrow buttons which control a moving window of two residues, highlighted in the Jmol viewer (Figure 7). The selected residue numbers can also be queried using the "?" button. Once the desired hinge location has been highlighted, the "Submit" button causes it to be recorded.

The user's job is queued upon submission. Each time an angle is varied over its full range by the Line Search minimizer, the user is emailed a progress notice. This process can be tracked and inspected using a viewer similar to that described in Figure 3. A few iterations later the job will converge and the user will receive a link to a viewer that animates the ligand binding motion. Links to structural coordinate files of the trajectory and the predicted holo structure are also provided.

## Discussion

### Prediction of protein conformational change

For RBP, ADK, GlnBP, and BC, large scale motions of the M domain are required for binding. For three of those, we successfully predicted the closed state within 6Å sRMSD. For BC, the *predicted *sRMSD was higher, but still about 4Å lower than for the *starting *structure, and with qualitative similarities to the *holo *(Figure 4).

### Ligands binding to the open conformation

MurA is a special case in which the ligand binds the *open *form. Our predicted structure was closed, with sRMSD *higher *than for its *starting *structure, by about 4Å. This is mostly because *f*, as a result of being trained on RBP, ADK, GlnBP, and BC for this protein, is explicitly biased towards closed structures through its *gyration_radius *term. If the *docked_energy *term were used alone in place of *f*, sRMSD would be much better, though still 0.6Å higher than for the *starting *structure (Table 1).

### Small, oxygen-rich ligands

The ligands SO_4 _and ribose docked deep inside one of the two domains of ADK and RBP (respectively), leading to poor sRMSD. SO_4_, ribose, and oxalic acid behaved similarly with GlnBP, leading to the highest sRMSD compared with other ligands (glutamine, leucine T6361, cAMP, ATP). This may be due to an insufficient desolvation penalty in the docking force field, or to the lack of explicit ions in the docking, at least for this class of ligands.

The above issue goes to the root of the utility of docking in predicting closed structures. We observed that the docked ligands in the predicted structures had relatively few receptor contacts in common with those in experimentally observed *holo *structures. And yet each of our five-fold leave-one-out studies verified the utility of the *docked_energy *term in *f*, for the purpose of detecting closed structures. It may be that the docking force field is biased to favor closed structures, in which the ligand can maximize the number of contacts in a non-specific manner. The cleft may also have non-specific ligand-binding properties [66]. This would explain the success of using various non-natural ligands to predict the closed form of GlnBP (table 2). It would also explain why CE performed well on RBP when ATP was used instead of its natural ligand (table 1). CE's performance on GlnBP, BC, and MurA was somewhat worse (~1.5Å on average) when ATP was substituted for the natural ligand, indicating at least some predicted interactions are specific.

## Conclusions

We conclude that for many hinge bending proteins it is possible to generate conformers similar to the closed structure given the apo. We note that due to shortcomings in the docking method, any small, oxygen-rich ligands should be replaced, and ATP is a good alternative. The hinge location was predicted using an existing hinge prediction server, and iterative rotations, equilibrations, and docking runs resulted in prediction of closed structures similar to those known crystallographically, in four of the five cases. The computational cost was moderate, permitting practical implementation on a single processor.

## Authors' contributions

SF conceived the idea, did the work, and wrote the manuscript. MG refined the idea, directed the work and provided critical reading and guidance on the manuscript. Both authors have read and approved the work.

## Supplementary Material

Additional file 1 **Implementation of the line search algorithm**. Starting from the apo structure, we generate conformations in the pitch, yaw, and roll rotational directions. After exploring in each direction, the conformer that minimizes *f *is the starting point for exploration in the next direction. The algorithm is converged when no rotation is possible in any direction that further minimizes *f*. * A particular direction is exhaustively explored when one conformer has been generated or attempted every 15° (±7.5°) in that direction, holding the other two direction angles constant (again ±7.5°). Click here for file
