# Supplementary figures and images for: Predicting protein ligand binding motions with the conformation explorer
Source: BMC Bioinformatics. 2011 Oct 27;12:417. doi: 10.1186/1471-2105-12-417 (PMC3354956; doi:10.1186/1471-2105-12-417)

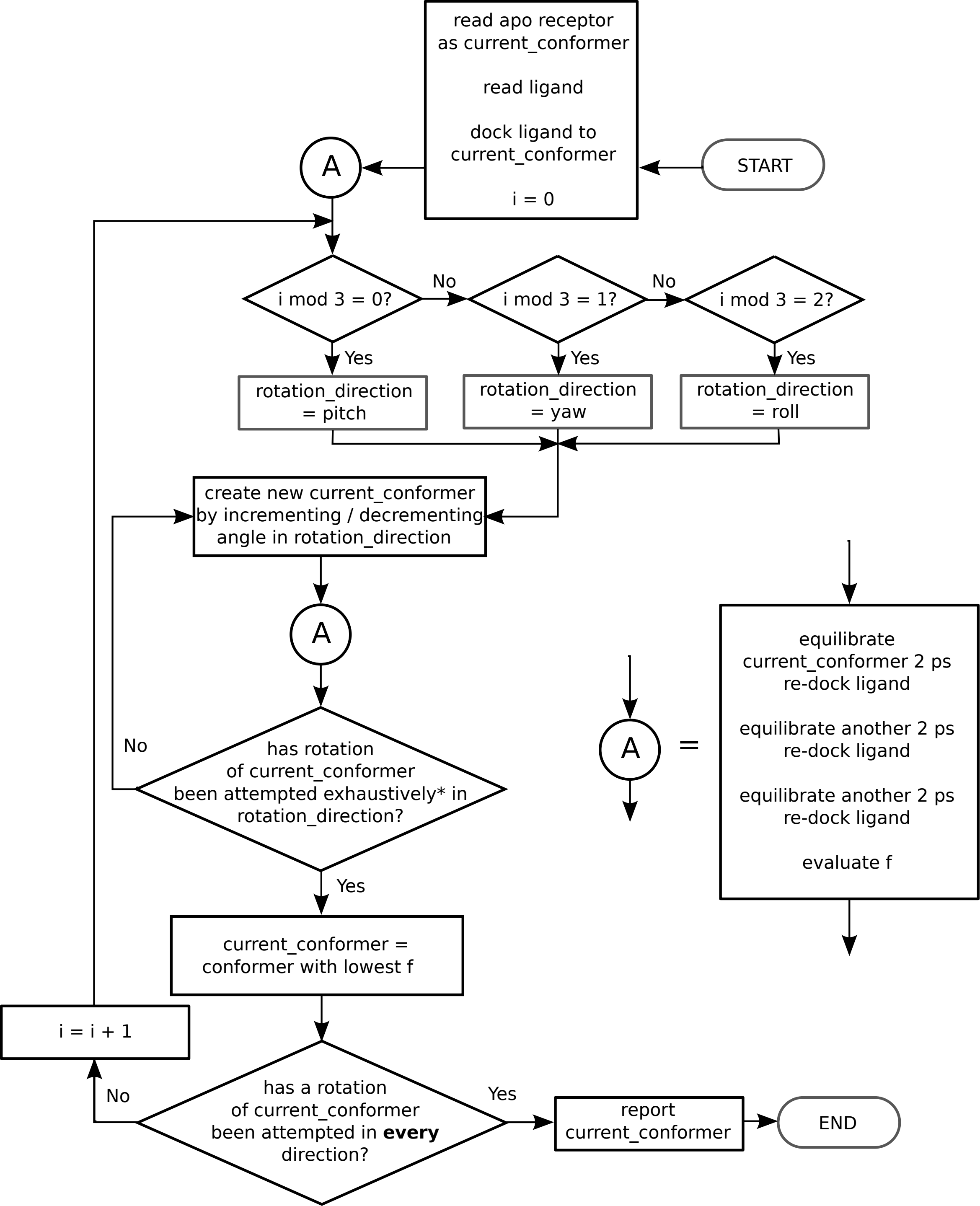

Supplement: Additional file 1 — Implementation of the line search algorithm. Starting from the apo structure, we generate conformations in the pitch, yaw, and roll rotational directions. After exploring in each direction, the conformer that minimizes f is the starting point for exploration in the next direction. The algorithm is converged when no rotation is possible in any direction that further minimizes f. * A particular direction is exhaustively explored when one conformer has been generated or attempted every 15° (±7.5°) in that direction, holding the other two direction angles constant (again ±7.5°). [file 1471-2105-12-417-S1.PDF]
